# A Mixture of Pure, Isolated Polyphenols Worsens the Insulin Resistance and Induces Kidney and Liver Fibrosis Markers in Diet-Induced Obese Mice

**DOI:** 10.3390/antiox11010120

**Published:** 2022-01-05

**Authors:** Hèctor Sanz-Lamora, Pedro F. Marrero, Diego Haro, Joana Relat

**Affiliations:** 1 Department of Nutrition, Food Sciences and Gastronomy, School of Pharmacy and Food Sciences, Food Torribera Campus, University of Barcelona, E-08921 Santa Coloma de Gramenet, Spain; h.sanz.lamora@ub.edu (H.S.-L.); pedromarrero@ub.edu (P.F.M.); 2 Institute for Nutrition and Food Safety Research, University of Barcelona (INSA-UB), E-08921 Santa Coloma de Gramenet, Spain; 3 Institute of Biomedicine, University of Barcelona (IBUB), E-08028 Barcelona, Spain; 4 CIBER Physiopathology of Obesity and Nutrition (CIBER-OBN), Instituto de Salud Carlos III, E-28029 Madrid, Spain

**Keywords:** antioxidants, food matrix, insulin resistance, kidney disease, obesity, polyphenols

## Abstract

Obesity is a worldwide epidemic with severe metabolic consequences. Polyphenols are secondary metabolites in plants and the most abundant dietary antioxidants, which possess a wide range of health effects. The most relevant food sources are fruit and vegetables, red wine, black and green tea, coffee, virgin olive oil, and chocolate, as well as nuts, seeds, herbs, and spices. The aim of this work was to evaluate the ability of a pure, isolated polyphenol supplementation to counteract the pernicious metabolic effects of a high-fat diet (HFD). Our results indicated that the administration of pure, isolated polyphenols under HFD conditions for 26 weeks worsened the glucose metabolism in diet-induced obese mice. The data showed that the main target organ for these undesirable effects were the kidneys, where we observed fibrotic, oxidative, and kidney-disease markers. This work led us to conclude that the administration of pure polyphenols as a food supplement would not be advisable. Instead, the ingestion of complete “whole” foods would be the best way to get the health effects of bioactive compounds such as polyphenols.

## 1. Introduction

Obesity is a worldwide epidemic with severe metabolic consequences. Obesity and its metabolic-related disorders are caused by various complex issues, one of which is an impairment of the adipose tissue functionality and expansion that result in an accumulation of lipids in organs such as liver, heart, pancreas, kidney, out of the white adipose tissue (WAT) [1,2] and an increase in circulating prooxidative, inflammatory adipokines [3,4]. 

The properties of bioactive compounds and the identification of new therapeutic targets have indicated their potential for the prevention and treatment of metabolic diseases such as obesity and its comorbidities [5]. Polyphenols are secondary metabolites in plants and the most abundant dietary antioxidants, which possess a wide range of health effects [5]. The most relevant food sources are fruit and vegetables, red wine, black and green tea, coffee, virgin olive oil, and chocolate, as well as nuts, seeds, herbs, and spices [6]. 

Polyphenols have been described as regulators of glucose homeostasis and insulin sensitivity by reducing hepatic glucose output, stimulating insulin secretion, and inhibiting glucose absorption in the intestines [7]. Furthermore, it has been suggested that the antioxidant capacity of polyphenols may protect against the reactive-oxygen-species (ROS)-related diseases such as insulin resistance, mitochondrial dysfunction, type 2 diabetes, inflammation [8]. Polyphenols have also been shown to induce apoptosis in cancer cells, which interferes with tumor generation and progression [9]. Furthermore, polyphenols’ effects have also been observed via lipid profile tests, inducing a hypolipidemic effect that reduced triglycerides as well as total and LDL cholesterol [10]. 

The bioavailability of polyphenols is low and can be modified by the attachment of additional functional groups onto their basic chemical structures (aglycons). Around 8000 structures of polyphenols have been described; basically, at least one phenolic ring with one or more hydroxyl groups attached can have a diverse physiological impact [11,12,13]. The absorption of polyphenols depends on the dose and the type, and their effects are associated with their bioavailability and pharmacokinetics [14]. In the intestinal tract, they have shown limited stability and a low absorption rate, likely related to the microbiome which transforms the ingested polyphenols to their active metabolites [15]. Once absorbed, polyphenols are metabolized after their arrival in the liver via portal circulation. This process has been shown to modify their structure and, as a result, their bioavailability and bioactivity [7,16]. Ultimately, the conjugated metabolites reach the bloodstream and the targeted tissues [7,16,17,18]. Moreover, the inclusion of polyphenols in a food matrix changes its bioavailability, safety, and biological activities [19,20,21]. Although most digestion assays have been done in vitro [22], other research has suggested that food matrices protect the bioactive compounds from intestinal degradation [20,23]. In addition, it has also been reported that exogenous supplementation with isolated bioactive compounds with antioxidant properties may be toxic [24]. 

Therefore, the aim of this work was to evaluate the ability of a pure, isolated polyphenol supplementation to counteract the pernicious metabolic effects of a high-fat diet (HFD).

For the design of the polyphenol mixture, we included at least one pure isolated compound from each group of the most consumed polyphenols included in the “Mediterranean diet” [25]. Synthetic flavanones, phenolic acids, stilbenes, and tyrosols were included, and a representative compound for each group was selected according to its ease of acquisition and its economic cost. As we were designing a hypothetical-yet-realistic food supplement, we needed to create one that was affordable, safe, and effective. 

The total amount of polyphenols added to the diet was calculated according to the polyphenol intake recommended as beneficial by the PREDIMED study, 820 mg in a human diet of 2300 kcal [25,26], and we calculated the dosage for the mice according to our previous research [27,28]. Detailed information about the nutritional intervention used is described below in the materials and methods section. 

Our results indicated that the administration of pure, isolated polyphenols within a HFD worsened the glucose metabolism in diet-induced obese mice. The data presented suggest that the main target organ for these undesirable effects were the kidneys, where we observed fibrotic, oxidative, and kidney-disease markers, thus indicating that the administration of pure isolated polyphenols as a food supplement would not be advisable. 

## 2. Materials and Methods

### 2.1. Animal Procedures: Dosage Regimen

All the procedures described in this paper were approved by the Animal Ethics Committee at the University of Barcelona (CEEA-UB-137/18P2). Four-week-old normoglycemic C57BL/6J littermate male mice were randomly divided into three groups: mice fed with a standard chow diet (Chow; Envigo 2918) (*n* = 8); mice fed with a 45% fat-derived-calories diet (HFD, D12451, Research Diets) (*n* = 11); and mice fed with a high-fat diet supplemented with pure, isolated polyphenols (HFD + Pol) (*n* = 10) (D12451, Research Diets D18060501). The HFD + Pol was prepared by Research Diets and included a representative mixture of pure, isolated polyphenols acquired from Sigma-Aldrich. This diet contained traces of (S)-2-[(Diphenylphosphino)methyl] pyrrolidine (0.014 mg/g diet). The composition of the polyphenol mixture was designed according to the research of Tresserra-Rimbau et al. [25], and it is detailed in Table 1. The dosage of the compounds included are in a similar and sometimes lower range compared to the dosages previously published for pure, isolated compounds. The usual range in previous publications goes from 15 mg/kg [29,30,31,32,33,34]. Considering mice of 35 g and an intake of 4.5 g/day of HFD that means for 15 mg/kg-0.525 mg of polyphenol and for 100 mg/kg-3.5 mg in mice. Cis-stilbene has been just used in cell culture for studying the role of polyphenols in proliferation and apoptosis and dosages are not comparable [35,36]. All of the compounds used, except for the cis-stilbene, have demonstrated previous beneficial effects in obesity and insulin resistance [5]. 

Animals were housed in a temperature-controlled room (22 ± 1 °C) on a 12 h/12 h light/dark cycle and were fed ad libitum. During the nutritional intervention, animals were weighed weekly, and diets were changed twice per week to prevent the oxidation of polyphenols. At the same time, food and beverage intake were recorded every two days. After 26 weeks of nutritional intervention, the animals were euthanatized by cervical dislocation. Blood was extracted by intracardiac puncture, and serum was obtained using centrifugation (1500 rpm, 30 min). Liver; subcutaneous white adipose tissue (scWAT); brown adipose tissue (BAT); and kidneys were isolated, immediately snap-frozen, homogenized with liquid nitrogen, and stored at −80 °C for future analysis.

The antioxidant capacity of the diets was measured at the beginning and the end of the nutritional intervention by the Folin–Ciocalteu method without any significant differences (data not shown). Folin–Ciocalteu is a colorimetric method based on the reduction of a mixture of phosphomolybdic and phosphowolframic acid (Folin–Ciocalteu Reagent 47641 Sigma-Aldrich, St. Louis, MO, USA) produced by polyphenols in an alkaline medium. In this redox reaction, tungsten and molybdenum oxides are formed, developing a blue color (absorbance measured at 765 nm) proportional to the total concentration of polyphenols.

### 2.2. Glucose-Tolerance Test (GTT) and Insulin-Tolerance Test (ITT)

To perform the GTT and ITT assays, the animals were transferred to clean cages and fasted for 6 h from 08:00 h (Zeitgeber Time 0) to 14:00 h (Zeitgeber Time 6) before the tests. A total of 1.5 mg glucose/g body weight (G7021, Sigma-Aldrich, St. Louis, MO, USA) for GTT, and 0.75 UI of insulin/kg body weight (Actrapid, Novo Nordisk, Bagsværd, Denmark) were injected intraperitoneally (i.p.) for GTT and ITT, respectively. Blood samples were collected from the tail vein of each mouse by gently massaging fourfold prior the injection (0 min) and at 30-, 60-, and 120-min post-injection. Glucose levels were measured using a glucometer (Glucocard SM, Menarini, Florence, Italy). GTTs were performed at weeks 7, 14, and 21, and ITTs at weeks 8, 15, and 22 of the nutritional intervention.

### 2.3. TBARS Assay

The lipid peroxidation was determined in 25 mg of homogenized kidney and liver, using TBARS kit (KA1381, Abnova, Taipei, Taiwan). This is a fluorometric kit that measures the content of malondialdehyde (MDA) at an excitation wavelength of 530 nm and an emission wavelength of 550 nm. The plates were read twice, and an MDA solution was used as a standard.

### 2.4. ELISA Assay

Lipocalin-2 serum levels were measured using EMLCN2 solid-phase sandwich enzyme-linked immunosorbent assay kit (ELISA) (EMLCN2 Thermo Fisher Scientific, Waltham, MA, USA). Absorbance was read at 450 nm, and a four-parameter standard curve (4PL) was performed using Graph Pad Prism 9.02. 

### 2.5. Triglycerides Quantification 

Liver tissue (100 mg) of each mouse were homogenized in 1 mL solution of Nonidet P40 at 5% (A1694, 0250, Panreac Applichem, Spain). The amount of TG was determined by using the Triglyceride Quantification Colorimetric Kit (MAK266, Sigma Aldrich, St. Louis, MO, USA). 

### 2.6. RNA Isolation and Quantitative Reverse Transcription PCR (qRTPCR) 

Total RNA was extracted from the previously homogenized frozen kidneys using TRI Reagent solution and Phasemaker tubes (A33250, Thermo Fisher Scientific, Waltham, MA, USA), followed by DNase I treatment (K2981, Thermo Fisher Scientific, Waltham, MA, USA) to eliminate genomic DNA contamination. The cDNA was synthetized from 1.5 µg of total RNA using a high-capacity cDNA reverse transcription kit (4368814, Applied Biosystems, Waltham, MA, USA). According to the measurement of the relative mRNA levels, quantitative (q) RT-PCR was performed using SYBR Select Master Mix for CFX (4472942, Applied Biosystems, Waltham, MA, USA) or TaqMan Gene Expression Master Mix (4369514, Applied Biosystems, Waltham, MA, USA). Each mRNA from a single sample was measured in duplicate, using M36b4 and B2m as housekeeping genes. The sequences of the primers used in the qPCR are presented in Supplemental Data (Table S1). Results were obtained by the relative standard curve method and expressed as fold increases, using the chow-diet experimental group as the reference.

### 2.7. Data Analysis/Statistics

Values were expressed as means ± SEM, and a *p*-value < 0.05 was considered statistically significant. Data were studied with statistical analyses using GraphPad Prism, version 9.02 (GraphPad, San Diego, CA, USA). The *p*-values were determined by using a one-way ANOVA with a follow-up Tukey’s test. When ANOVA tests presented different variances, Brown–Forsythe, and Welch’s corrections with a follow-up Dunnett’s T3 tests were applied. For repeated measures the *p*-values were calculated by using a 2-way ANOVA with Geisser–Greenhouse correction. Then a Tukey’s multiple comparison tests were performed between groups for each week. 

## 3. Results

### 3.1. Pure, Isolated Polyphenol Mixture Significantly Enhances the HFD-Induced Hyperphagia

**Figure 1:**
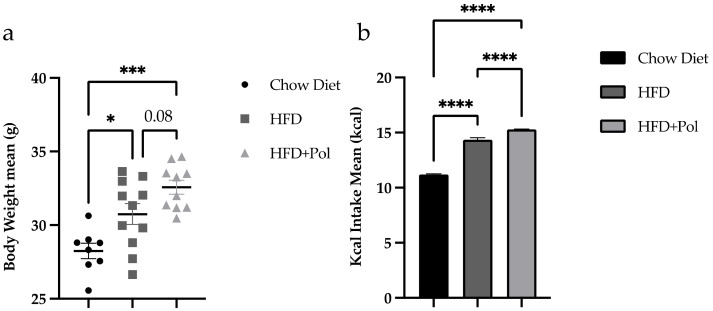
Polyphenol dietary supplementation increased the Kcal intake of HFD-only mice. (**a**) The graph represents the weight-gain mean between the beginning and the end of the dietary intervention (26 weeks). (**b**) The graph represents the food-intake average (Kcal) during the nutritional intervention. Data are presented as the mean ± SEM. * *p* < 0.05; *** *p* < 0.001; **** *p* < 0.0001. The *p*-values were determined by using a one-way ANOVA test and a Tukey’s multiple tests correction. Chow Diet *n* = 8; HFD = 11; HFD + Pol = 10.

HFDs have been shown to induce obesity due to their high energy density from fat and increased food intake, as compared to standard normocaloric diets [37]. In this study, it was observed that, as expected, HFD increased the body weight in both experimental groups (Figure 1). Comparing HFD-only mice with HFD + Pol mice, the dietary supplementation of an HFD with pure, isolated polyphenols had produced a significant increase in kcal intake (Figure 1b) but none in the animal body weight, even an upward trend was observed (*p* = 0.08) (Figure 1a). The weekly body weight and food intake are shown in supplemental figures (Figure S1a,b respectively). 

**Figure 2:**
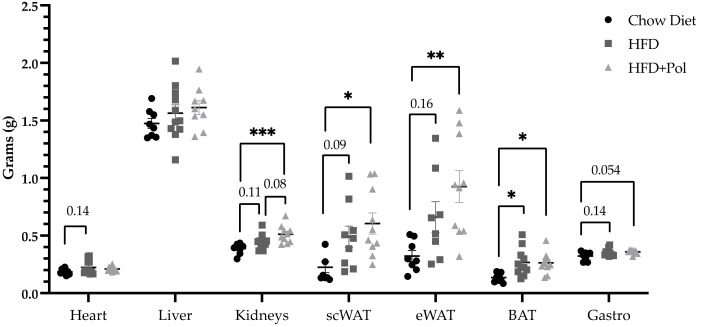
The polyphenol supplementation produced extra weight gain in HFD + Pol murine kidneys, as compared to HFD-only murine kidneys. The graph represents the tissue weight (g) of different tissues. Data are presented as the mean ± SEM. * *p* < 0.05; ** *p* < 0.01; *** *p* < 0.001. The *p*-values were determined by using a one-way ANOVA test and a Tukey’s multiple tests correction. Chow Diet *n* = 8; HFD = 11; HFD + Pol = 10.

Despite there were no differences in the weight gain between HFD-only mice and HFD + Pol mice, we evaluated the tissue weights to determine if there were differences due to the dietary nutritional intervention. As can be seen in Figure 2, subcutaneous (sc) and epididymal (e) WAT, BAT, and kidneys exhibited an upward trend in HFD-only mice compared to control mice and a significant increase in HFD + Pol mice. Moreover, in the case of the kidneys this tendency is also observed in the HFD + Pol when compared to HFD-only mice. 

### 3.2. Mice Supplemented with Polyphenols Show a Worse Response to Glucose and Insulin Bolus than HFD-Only Mice

**Figure 3:**
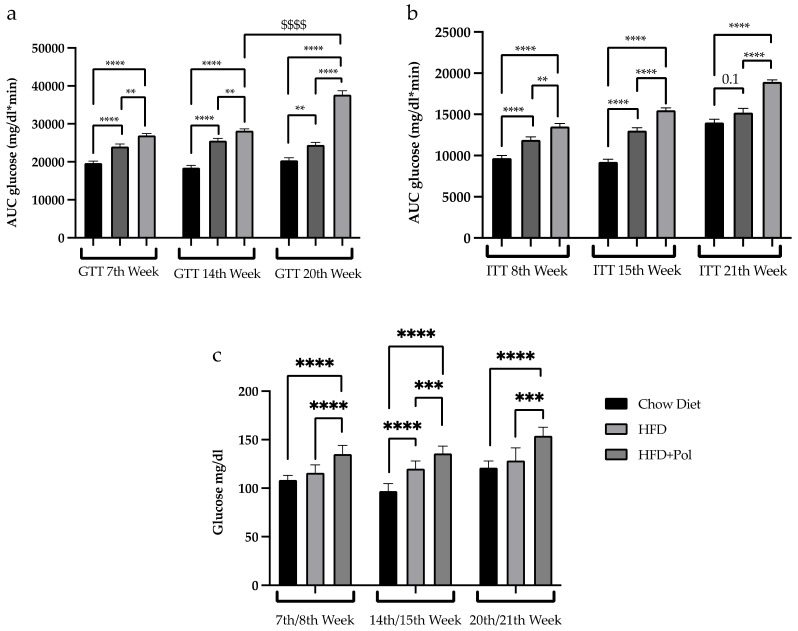
Polyphenol dietary supplementation worsened the glucose metabolism caused by an HFD. (**a**) AUC of the plasma glucose levels after i.p. administration of glucose (1.5 g/kg body weight (b.w.)) in standard-chow-diet, HFD, and HFD + Pol mice groups from the GTTs performed at weeks 7, 14, and 20; (**b**) AUC of the plasma glucose levels after i.p. administration of insulin (0.75 UI/kg b.w) in standard chow-diet, HFD, and HFD + Pol mice groups from the ITTs performed at weeks 8, 15, and 21; (**c**) Fasting blood glucose levels after 6 h of fasting. Data are presented as the mean ± SEM. ** *p* < 0.01; *** *p* < 0.001; **** *p* < 0.0001. $$$$ *p* < 0.0001. The *p*-values for each week were determined by using a one-way ANOVA test and a Tukey’s multiple tests correction. Chow Diet *n* = 8; HFD = 11; HFD + Pol = 10.

As expected, the HFD produced insulin resistance and glucose intolerance. In our experimental model, this impairment in glucose metabolism was observed from week 7 for the GTT and week 8 for the ITT (Figure 3a,b). Our data indicated that the polyphenol supplementation worsened the insulin resistance and glucose intolerance caused by the HFD. The polyphenol-supplemented mice exhibited a worse response to glucose and insulin during the nutritional intervention, as is demonstrated by the GTT and ITT curves (Figures S1 and S2, respectively). This corresponded to a significant increase in the AUCs of both GTT (Figure 3a) and ITT (Figure 3b), indicating a progressive aggravation of the insulin and glucose responses. 

This worsened response to glucose and insulin bolus was paired with higher fasting blood glucose in the polyphenol-supplemented animals, as compared to the HFD-only mice (Figure 3c). Contrary to what was expected, our data indicated that the administration of pure, isolated polyphenols added directly to a HFD did not produce healthy benefits but increased the HFD-induced insulin resistance.

### 3.3. Polyphenol-Supplemented HFD Increases the Oxidative Stress Markers in the Kidney

The slight increase in the absolute weight of the kidneys between HFD-only mice and HFD + Pol mice would encourage us to analyze the kidneys of these animals more deeply. 

The kidney is one of the tissues targeted by obesity and insulin resistance. Pathologies such as chronic kidney disease (CKD) and diabetic nephropathy are caused not just by high glucose circulating levels but also by lipid accumulation in the renal tissue that contributes to the development of glomerulitis, chronic inflammation, a high production of ROS, and fibrosis [38]. In this situation, the damaged kidney activates the renin–angiotensin–aldosterone system (RAAS) and secretes specific cytokines that aggravate the systemic symptomatology associated to the CKD and diabetic nephropathy such as hypertension, cardiovascular risk [39,40]. 

To evaluate the kidney condition, we firstly evaluated the oxidative stress through a thiobarbituric acid reactive substances (TBARS) assay. TBARS quantifies the levels of malondialdehyde (MDA) produced by the decomposition of the unstable peroxides. A TBARS assay can be used as a measurement of damage caused by oxidative stress [41]. 

Moreover, the expression of antioxidant defense enzymes *glutathione s-reductase (Gsr), catalase (Cat),* and *superoxide dismutase (Sod)* was measured to evaluate the kidney response to oxidative stress. 

**Figure 4:**
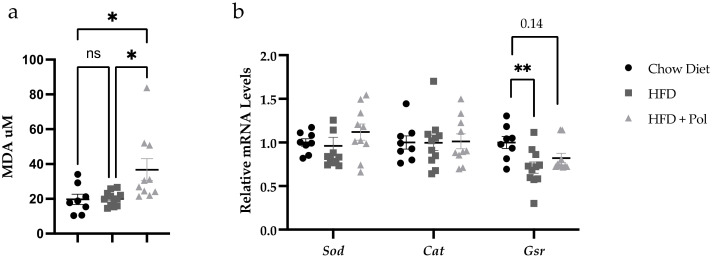
Polyphenol-supplemented HFD increased the oxidative stress markers in the kidneys. (**a**) Malondialdehyde (MDA) levels in the kidneys of standard-chow-fed, HFD-only, and HFD + Pol mice measured by the TBARS assay. (**b**) Relative mRNA levels of *Sod, Cat,* and *Gsr*. Data are presented as the mean ± SEM. * *p* < 0.05; ** *p* < 0.01. The *p*-values were determined by using *a* one-way ANOVA test and a Tukey’s multiple tests correction. Chow Diet *n* = 8; HFD = 11; HFD + Pol = 10.

The levels of malondialdehyde (MDA) were increased in the kidneys of the HFD + Pol mice (Figure 4a). Regarding the relative mRNA levels of antioxidant-defense genes, the data showed a significant reduction in the relative mRNA levels of *Gsr* and no significant changes in the expression of *Sod* and *Cat* in HFD-only mice (Figure 4b). No significant changes neither with control mice nor HFD-only mice were detected in HFD + Pol mice. 

### 3.4. Polyphenol-Supplemented HFD Upregulates the Expression of Fibrosis and Kidney Damage Markers 

**Figure 5:**
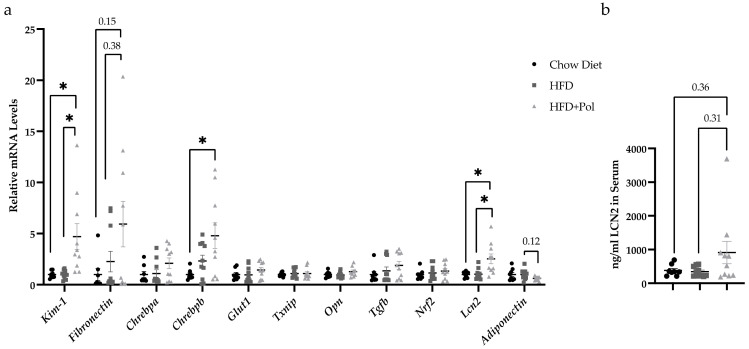
HFD + Pol upregulated the expression of fibrosis and kidney-damage markers. (**a**) Relative mRNA levels of several fibrosis, oxidative stress and kidney damage markers, *kidney injury molecule-1(kim1), fibronectin-1, carbohydrate-responsive element-binding protein b* *(Chrebpb), glucose transpoorter1 (Glut1), thioredoxin-interacting protein (Txnip), Osteopontin (Opn), Transforming growth factor beta-1 (Tgfb), Nuclear factor erythroid 2-related factor 2 (Nrf2), Lipocalin 2 (Lcn2)* and *Adiponectin*. (**b**) Protein levels of LCN2 measured by ELISA in the kidney. Data are presented as the mean ± SEM. * *p* < 0.05. The *p*-values for the ELISA assay and *Glut1, Txnip, Opn, Tgfb* and *Nrf2* genes were determined by using a one-way ANOVA test and Tukey’s multiple tests correction. For genes showing different variances like *Kim-1, Fibronectin-1, Cherbpa, Cherbpb, Lcn2* and *Adiponectin* a one-way ANOVA with Brown-Forsythe and Welch’s correction and a Dunnett’s comparisons tests were performed. Chow Diet *n* = 8; HFD = 11; HFD + Pol = 10.

The onset and progression of CKD can be analyzed by the measurement of fibrosis, oxidative stress, and kidney damage markers. Thus, an expression profile of fibrosis and kidney damage markers was conducted. Our results showed an upregulation of *kidney injury molecule-1 (Kim-1)* [42], and an upregulation in the mRNA levels of the transcription factor *carbohydrate-responsive element-binding protein b (Chrebpb)*, the increase of which has been related to the progression of diabetic kidneys (Figure 5a) [43]. In addition, an upward trend in the expression of *fibronectin (Fn-1)*, a protein related to fibrotic processes [44,45] was also detected. 

The HFD + Pol mice also showed a significant upregulation of *lipocalin-2 (lcn2)* (Figure 5a). LCN2 circulating levels increase under different pathological states, particularly kidney injury, bacterial infection, and inflammation as well as in people of advanced age. LCN2 is a biomarker for the development of renal injury, and it is considered an acute phase protein when upregulated in the kidney tubules [46,47,48]. We also measured the protein levels of LCN2 in the kidneys and despite no significant results a clear upward trend was observed in the kidneys of the HFD + Pol mice (Figure 5b). 

### 3.5. Polyphenol-Supplemented HFD Upregulates the Expression of Fibrosis Markers in the Liver and Increases the Hepatic Lipid Content

The liver is a key organ in the maintenance of metabolic homeostasis and is one of the main organs affected by the accumulation of ectopic lipids. The nonalcoholic fatty liver disease (NAFLD) has been strongly associated with obesity and insulin resistance, as well as type 2 diabetes [49,50]. It is well-known that the accumulation of intrahepatic fat leads to liver steatosis that is an important factor for the metabolic complications associated with obesity [51]. To evaluate the impact of dietary polyphenols supplementation in hepatic steatosis, we measured the TG content in the livers of HFD-only mice and HFD + Pol mice the expression of different genes to define the general state of these livers HFD + Pol. 

**Figure 6:**
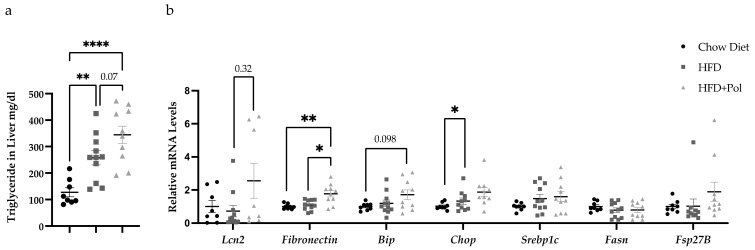
HFD + Pol upregulated the expression of fibronectin in the liver. (**a**) Hepatic TG content. The concentration of TG (ng/uL) was measured in the livers of chow, HFD and HFD + Pol mice. (**b**) Relative mRNA levels of different genes to evaluate the general state of the livers, *fatty acid synthase (Fasn), sterol regulatory element binding protein, (Srebp1c), Cell death activator CIDE-3, (Fsp27)*, *Binding immunoglobulin protein (Bip)* and *C/EBP Homologous Protein (Chop).* (**b**) Protein levels of LCN2 measured by ELISA in the kidney. Data are presented as the mean ± SEM. * *p* < 0.05; ** *p* < 0.01; **** *p* < 0.0001. The *p*-values for the TG assay and *Fasn* gene were determined by using a one-way ANOVA test and a Tukey’s multiple tests correction. For genes showing different variances like *Fsp27B, Fibronectin-1, Srebp1c, Chop, Lcn2* and *Bip* a one-way ANOVA with Brown-Forsythe and Welch’s correction and a Dunnett’s comparisons tests were performed. Chow Diet *n* = 8; HFD = 11; HFD + Pol = 10.

As it is showed in Figure 6a, the hepatic lipid content is higher in both groups of HFD-fed mice compared to control mice and, despite no significance, an upward trend is observed in the HFD + Pol mice compared to HFD-only mice (*p* = 0.07). Regarding the different markers evaluated, our results showed that *fibronectin (Fn-1)* is upregulated in HFD + Pol mice (Figure 6b), thus suggesting a fibrotic process in the livers of polyphenol-supplemented mice. 

Besides fibrosis, we also analyzed the expression of genes related to *de novo* lipogenesis *(fatty acid synthase (Fasn)* and *sterol regulatory element binding protein, (Srebp1c)),* and lipid droplets formation (*Cell death activator CIDE-3, (Fsp27b)*), but also markers of the reticulum stress (*Binding immunoglobulin protein (Bip) and C/EBP Homologous Protein (Chop))*. No changes in the mRNA levels were detected in any of the genes analyzed (Figure 6b).

## 4. Discussion

Our data demonstrated that the supplementation of an HFD with pure, isolated polyphenols worsened the effects of the HFD by producing a homeostatic imbalance that resulted in renal and liver fibrosis in mice. We concluded that the HFD + Pol mice exhibited a dysregulation in glucose and insulin metabolism and signs of kidney damage due to their increased levels of MDA, which suggested higher oxidative stress (Figure 4), as well as the results of their gene-expression analyses (Figure 5), where the changes observed in HFD + Pol mice was related to CKD and obesity [52,53,54,55,56], and liver fibrosis. 

The kidney damage in HFD + Pol mice was basically defined by the upregulation of *Kim-1* and *Lcn2*. 

KIM-1 is a transmembrane glycoprotein with a low expression in kidney but significantly upregulated in damaged kidneys. KIM-1 upregulation in CKD has been associated with an hypoxic environment [57]. A chronic hypoxia due to the structural and functional disorders (alteration of the capillarity, excessive activity of renin-angiotensin system, oxidative stress…) in the kidney is the main pathogenic mechanism of progressive CKD. Hypoxia is a powerful stimulus for KIM-1 expression in the proximal tubular cells. The upregulation of KIM-1 increases the release of cytokines and chemokines, that enhances inflammation, hypoxia and fibrosis, thus aggravating the CKD [58,59]. 

Based on the literature, one of the possible links between renal damage and the general decline in glucose metabolism could be the iron metabolism. Due to the role of Lcn2 in the iron metabolism, we also measured the mRNA levels of *transferrin receptor 2 (trf2), hephaestin (heph),* and *hepcidin (hamp)* in the liver and in the kidney (data not shown), but no differences between the HFD + Pol and HFD-only mice were detected. Similarly, neither the circulating levels of iron were different in supplemented mice versus HFD-fed animals (data not shown). It has been documented that LCN2 is an iron-carrier protein, and its biological activity depends on its iron-load and on where it is produced (renal tubules or macrophages), which defines its dual role in the development of kidney damage [60,61,62]. Iron-free LCN2 secreted by renal tubular epithelial cells has been associated with renal injury, and the expression and release of macrophage-derived iron-bound LCN2 has been linked to renal recovery [63]. Similarly, it has also been shown that adipose-tissue-derived LCN2 plays a critical role in causing both chronic and acute renal injury, but it is also essential for the progression of CKD in rodent and human models [46,64]. Viau et al. concluded that LCN2 may act as a growth regulator by mediating the mitogenic effects of epidermal growth factor receptor (EGFR) signaling [46]. The activation of EGFR has been linked to the regulation of several other cellular responses involved in the progression of renal damage, including cell proliferation, inflammatory processes, and extracellular matrix regulation [65]. In our experimental study, the levels of Lcn2 were unaltered in the adipose tissue of the HFD + Pol mice (data not shown). 

Fibrosis was evaluated by measuring the mRNA levels of fibronectin both in the kidney and in the liver. Fibrosis is caused by a pathological excess of extracellular matrix deposition that leads to a disruption of tissue structure that at the end should provoke a loss of function. The fibrotic process involves a complex network of signal transduction pathways where the transforming growth factor-beta (TGFb) has a central role [45]. Fibrosis is determined among others by an increase in the expression of collagens, proteoglycans, glycoproteins, and fibronectins [44]. Fibronectin is one of the main players by affecting the TFGb release and as responsible protein for the accumulation of collagen and hence the development of fibrosis [66,67]. 

Altogether, our results suggested that polyphenol-supplementation within a HFD drives to the development of fibrosis in the liver and kidneys that could aggravate the insulin resistance in DIO mice. 

### The Importance of Food Matrices on the Effects of Polyphenol-Supplementation

Our results indicated a pernicious effect of pure, isolated polyphenols when administered under HFD conditions. These results may be controversial given current and ongoing research, but we believe that all aspects of polyphenol supplementation should be discussed. In 2010, Bouayed and Torsten published a review suggested the “double-edged sword” of the cellular redox state and exogenous antioxidants [24]. Their report as well as recent research have indicated that it is essential to consider the type, the dosage, the combination, and the consumption matrices involved when using bioactive compounds as the administration of such may alter the physiological balance between oxidation and antioxidation pathways, which may result in either beneficial or deleterious effects [68,69]. 

The inclusion of complete foods naturally rich in antioxidants (e.g., fruits and vegetables) has been widely recommended by health organizations and has been the basis of many “healthy eating” programs, such as the Mediterranean diet [70,71]. Much research has been focused on the antioxidant effects of phenolic compounds when administered in isolation versus when ingested via their natural source [5], and increasing data has suggested that the beneficial properties of complete foods cannot be attributed to a single compound. Rather, it is the consumption of the food in its whole state (i.e., not extracted, or isolated compounds) that activates the additive, synergistic, and antagonistic effects of the phytochemicals and nutrients. The effects of polyphenols depend on their bioavailability, and it is assumed that just the 5–10% of the total dietary polyphenol intake is absorbed directly through the stomach and/or small intestine [12]. The rest of ingested polyphenols reaches the colon where they are transformed by the microbiota [72,73]. When absorbed, polyphenols undergo phase I and II metabolism (sulfation, glucuronidation, methylation, and glycine conjugation) in the liver [14]. The new-synthesized metabolites may then impact, among others, in the adipose tissue, pancreas, muscle, and liver, where they exert their bioactivity [5].

In addition, it is not only the natural food matrices that are essential [74,75], but also the preparation process and the conditions under which it is ingested (e.g., cooking, juicing, etc.). It has been widely demonstrated that cooking affects the phytochemical content of foods as well as their chemical structures and the bioavailability of their bioactive compounds; in other words, how a food is processed before consumption may be directly related to its health effects [76,77,78,79,80,81]. 

## 5. Conclusions

In conclusion, our work demonstrated that the dietary supplementation of an HFD with pure, isolated polyphenols worsened the metabolic disturbances known to be caused by HFDs and directly impacted the kidneys and the liver, increasing oxidative stress, renal damage, and fibrosis. The data presented reinforced the recent trends eschewing pure, isolated antioxidant replacements and instead encouraging the ingestion of complete whole foods, from which the beneficial bioactive compounds originated. 

### Limitations and Follow-Up

This is an initial work that opens a new research line to study the impact of pure isolated polyphenols in the onset and development of obesity and its metabolic-related pathologies such as insulin resistance and NAFLD. We are aware that the study has limitations, and that further analysis are needed. According to the data presented the next step would be to identify the role of each compound individually on the effects described. A follow-up study is underway to analyze the effects of each compound individually in culture cells to evaluate which compounds or compounds could be the responsible for the deleterious effects observed or if it is the complete mixture. Moreover, a pair-fed study to disassociate the effect of polyphenols from increased caloric intake per se should be a follow-up. 

To calculate the power analysis of this experiment our main outcome is the insulin resistance caused by a HFD. Considering the results showed in Figure 2 our experimental approach reaches the significance expected. We have positive results in the kidney and in the liver which means that our experimental approach is well designed, but some variables show a high intragroup variability mainly in the HFD-supplemented animals. This variability is probably due to differences in the polyphenol absorption and bioavailability. 

## Figures and Tables

**Table 1:** Composition of polyphenol mixture.

Family	% Total Polyphenols	Polyphenol (Cas Num)	mg/g Diet
Flavanones	54.4%	Hesperidin (520-26-3)	0.738
		(±)-Naringenin (67604-48-2)	0.234
Phenolic Acids	35.3%	2-Hydroxycinnamic acid (614-60-8)	0.594
		Syringic acid (530-57-4)	0.036
Stilbenes	1.2%	cis-Stilbene (645-49-8)	0.022
Tyrosols	9.1%	Tyrosol (510-94-0)	0.162
Total	100%		1.786

## Data Availability

The data presented in this study are available in this manuscript.

## Supplementary Materials

The following are available online at https://www.mdpi.com/article/10.3390/antiox11010120/s1, Table S1: Sequences of the primers used in SYBR green assays and references for the probes used in TaqMan assays. Figure S1: The curves of GTTs. Figure S2: The curves of ITTs.
