# Supplementary material for: A Mixture of Pure, Isolated Polyphenols Worsens the Insulin Resistance and Induces Kidney and Liver Fibrosis Markers in Diet-Induced Obese Mice
Source: Antioxidants (Basel). 2022 Jan 5;11(1):120. doi: 10.3390/antiox11010120 (PMC8772794; doi:10.3390/antiox11010120)

**Table S1:** Sequences of the primers used in SYBR green assays and references of the probes used in TaqMan assays.

| Primers | Forward | Reverse |
|---|---|---|
| *B2m* | ACTGATACATACGCCTGCAGAGTT | TCACATGTCTCGATCCCAGTAGA |
| *M36b4* | AGATGCAGCAGATCCGCAT | GTTCTTGCCCATCAGCACC |
| *Sod* | AGCTCATCTCAGAGCTCGTCCG | AGCTCATCTCAGAGCTCGTCCG |
| *Cat* | GTCACTCAGGTGCGGACATT | CAGGGTGGACGTCAGTGAAA |
| *Gsr* | CAGTTGGCATGTCATCAAGCA | CGAATGTTGCATAGCCGTGG |
| *Kim-1* | CTGCTGCTACTGCTCCTTGT | GGAAGGCAACCACGCTTAGA |
| *Fibronectin* | GGAACCAGCAGAGTCCCAAA | ACACCCAGCTTGAAGCCAAT |
| *Chrebpa* | CGACACTCACCCACCTCTTC | TTGTTCAGCCGGATCTTGTC |
| *Chrebpb* | TCTGCAGATCGGTGGAG | CTTGTCCCGGCATAGCAAC |
| *Glut1* | GCTTCCTGCTCATCAATCGTAAC | CATCGGCTGTCCCTCGAA |
| *Txnip* | GTCAGTGTCCCTGGCTCCAAGA | AGCTCATCTCAGAGCTCGTCCG |
| *Opn* | TTCTCCTGGCTGAATTCTGAGG | AATCAGTCACTTTCACCGGG |
| *Tgfb* | ACGTCACTGGAGTTGTACGG | GGGCTGATCCCGTTGATTTC |
| *Nrf2* | CAGTGGATCCGCCAGCTAC | GGCAAGCGACTCATGGTCATC |
| *Lcn2* | GGACCAGGGCTGTCGCTACT | GGTGGCCACTTGCACATTGT |
| *Adiponectin* | CAGTGGATCTGACGACACCAA | TGGGCAGGATTAAGAGGAACA |

**Figure S1:** GTT curves showing plasma glucose levels after i.p. administration of glucose (1.5 g/kg b.w) in Chow diet, HFD and HFD + Pol fed mice after 7, 14 and 20 weeks of polyphenol-supplementation. Data are presented as the mean ± SEM. # p<0.05; ## p<0.01; ### p<0.001 versus Chow diet-fed animals; * p<0.05; ** p<0.01 versus HFD-fed animals (n=8-11/group).

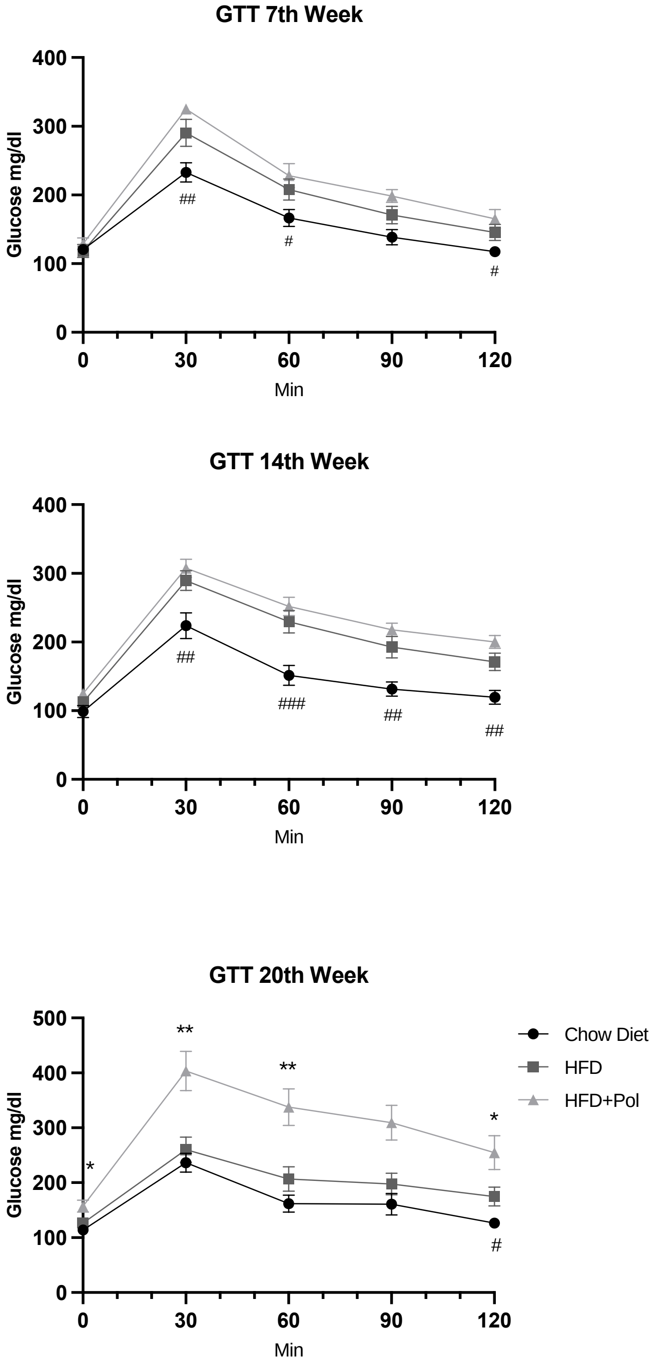

**Figure S2:** ITT curves showing plasma glucose levels after i.p. administration of insulin (0.5 UI/kg b.w) in Chow diet, HFD and HFD + Pol fed mice after 8, 15 and 21 weeks of polyphenol-supplementation. Data are presented as the mean ± SEM. # p<0.05; ## p<0.01 versus Chow diet-fed animals; * p<0.05 versus HFD-fed animals (n=8-11/group).

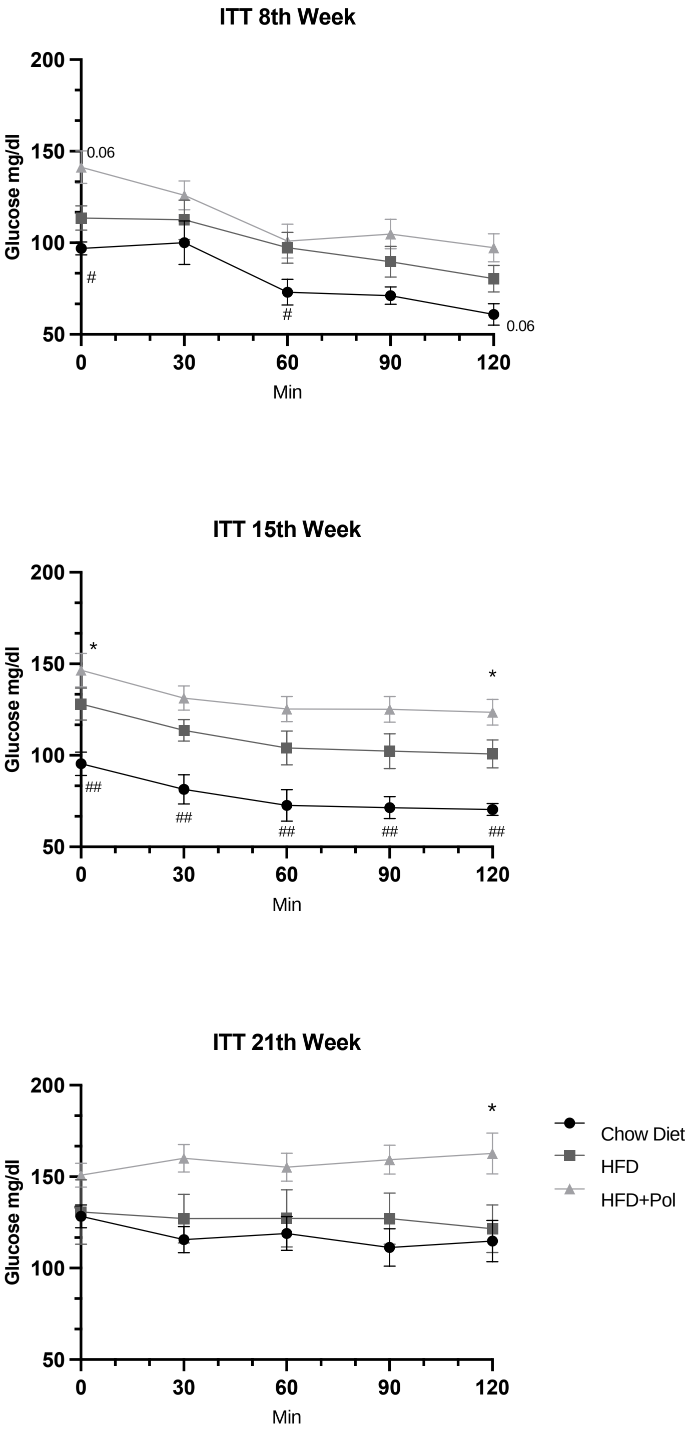

Supplement: Supplementary file 1 [file antioxidants-11-00120-s001.zip › antioxidants-1469506-supplementary.pdf]
